# DBSCAN and DBCV application to open medical records heterogeneous data for identifying clinically significant clusters of patients with neuroblastoma

**DOI:** 10.1186/s13040-025-00455-8

**Published:** 2025-06-12

**Authors:** Davide Chicco, Luca Oneto, Davide Cangelosi

**Affiliations:** 1 Università di Milano-Bicocca, Milan, Italy; 2 University of Toronto, Toronto, Ontario, Canada; 3 Università di Genova, Genoa, Italy; 4 IRCCS Istituto Giannina Gaslini, Genoa, Italy

**Keywords:** Neuroblastoma, Electronic health records, EHRs, Childhood cancer, Open data, Unsupervised machine learning, DBSCAN, DBCV, Clustering

## Abstract

Neuroblastoma is a common pediatric cancer that affects thousands of infants worldwide, especially children under five years of age. Although recovery for patients with neuroblastoma is possible in 80% of cases, only 40% of those with high-risk stage four neuroblastoma survive. Electronic health records of patients with this disease contain valuable data on patients that can be analyzed using computational intelligence and statistical software by biomedical informatics researchers. Unsupervised machine learning methods, in particular, can identify clinically significant subgroups of patients, which can lead to new therapies or medical treatments for future patients belonging to the same subgroups. However, access to these datasets is often restricted, making it difficult to obtain them for independent research projects. In this study, we retrieved three open datasets containing data from patients diagnosed with neuroblastoma: the Genoa dataset and the Shanghai dataset from the Neuroblastoma Electronic Health Records Open Data Repository, and a dataset from the TARGET-NBL renowned program. We analyzed these datasets using several clustering techniques and measured the results with the DBCV (Density-Based Clustering Validation) index. Among these algorithms, DBSCAN (Density-Based Spatial Clustering of Applications with Noise) was the only one that produced meaningful results. We scrutinized the two clusters of patients’ profiles identified by DBSCAN in the three datasets and recognized several relevant clinical variables that clearly partitioned the patients into the two clusters that have clinical meaning in the neuroblastoma literature. Our results can have a significant impact on health informatics, because any computational analyst wishing to cluster small data of patients of a rare disease can choose to use DBSCAN and DBCV rather than utilizing more common methods such as *k*-Means and Silhouette coefficient.

## Introduction

Neuroblastoma is a childhood cancer that affects approximately 6,000 infants and contributes to 15% of cancer-related deaths in children worldwide. Neuroblastoma forms in the tissues of human nerves, is the most prevalent extracranial solid tumor in children, and usually affects patients under five years old [1]. The survival rate for kids with high-risk neuroblastoma is only 40%, while it can reach 80% for low-risk neuroblastoma. Medical treatments for patients with neuroblastoma include surgery, chemotherapy, radiation therapy, and immunotherapy [1].

Scientific research can help medical doctors understand the development and progression of the disease and can be conducted in several ways. Bioinformatics and computational biology research on genomics data, for example, can help unveil the genes most involved in neuroblastoma diagnosis and prognosis [2–7]. On the other hand, data derived from electronic health records (EHRs), collected after hospital laboratory exams (such as blood tests), can be an useful asset for computational analyses [8].

In the past, researchers applied computational statistics and machine learning to several datasets of EHRs of patients with neuroblastoma.

Regarding machine learning, we performed a computational intelligence study on the data of Italian Registry of Peripheral Neuroblastoma (RINB) [9], where we applied a supervised machine learning approach to detect the most predictive clinical variables for the outcome of patients with neuroblastoma. In that case, however, the data could not be released publicly for privacy reasons.

Sometimes, fortunately, study authors are allowed to release EHRs data of patients with neuroblastoma openly online for free, without any restrictions. Barbara Banelli and colleagues [10] investigated the role of 17 genes of the Protocadherin B cluster (PCDHB) on a dataset of genomics and EHRs of 121 patients with stage-4 neuroblastoma. They applied computational statistical methods for a survival analysis on this dataset, by employing the SPSS proprietary software. SPSS is the programming language employed also by Yangyan Ma and coauthors [11] in their study, where they analyze a dataset of EHRs and genetics information to identify the most relevant prognostic factors for 169 patients with stage-3 or stage-4 neuroblastoma.

Shunsuke Kimura et al. [12] analyzed a subset of data from the well-known TARGET-NBL project [13, 14]. They used both genetics data and EHRs to perform a computational statistical analysis through the R open source programming language. The main outcomes of this study regard genomics and the roles of some genes (or gene clusters) over others.

All these three studies have a particular asset: their authors released their neuroblastoma dataset openly and for free, so that it could be analyzed by anyone else in the world, following the FAIR principles [15]. We cleaned the dataset of Barbara Banelli and colleagues [10] and the dataset of Yangyan Ma and coauthors [11], and we described them thorougly and released them in our Neuroblastoma Electronic Health Records Open Data Repository [16, 17], as *dataBB2013* and *dataYM2018* respectively.

Unsupervised machine learning methods, such as clustering, applied on data of EHRs can identify clinically significant groups of patients based on their medical features. These clusters, in turn, can be useful to identify significant subgroups of patients that need particular treatments or therapies.

In the present study, we decided to apply several clustering algorithms to the Genoa dataset of Barbara Banelli and colleagues [10], to the Shanghai open dataset of Yangyan Ma and coauthors [11], and to a subset of the TARGET-NBL stage-4 dataset of Shunsuke Kimura et al. [12]. Among the 94 patient profiles of that dataset, we removed three rows having unknown diagnostic category, and kept only the 93 rows with diagnostic category equal to neuroblastoma or nodular ganglioneuroblastoma, which is a variant of neuroblastoma surrounded by ganglion cells. We describe in detail this dataset in “Datasets” section.

Other resources for EHRs data of patients diagnosed with pediatric neuroblastoma exist. The International Neuroblastoma Risk Group (INRG) has released and currently maintains the INRG Data Commons [18, 19], launched within the Pediatric Cancer Data Commons [20, 21], a global data collection initiative coordinated by University of Chicago (Chicago, Illinois, USA). The INRG Data Commons is a database of thousands of EHRs of patients with this oncological disease, but its access is restricted: researchers who want to analyze these data need to submit a proposal, that needs to be evaluated by an INRG committee who might approve it or not.

On the contrary, the Genoa, Shanghai, and TARGER-NBL datasets analyzed in this study are completely open, unrestricted, public, and can be be analyzed by anyone worldwide.

When high-quality health datasets are available, they can be analyzed using either supervised or unsupervised methods. Supervised approaches are employed when a gold standard piece of information is available, while unsupervised learning methods are used when there is no clear ground truth. Unsupervised problems are more complex but often more useful for investigation in medical sciences. In fact, patients frequently arrive at the hospital without clear information regarding their prognosis, diagnosis, or condition. This context is more appropriately framed within an unsupervised framework rather than a supervised one. Cutting-edge biomedical research is unsupervised.

Clustering algorithms are unsupervised computational methods which can split data into significant groups, called *clusters*, that otherwise would probably be unnoticeable to human beings. The research question we investigate in this study concerns the efficacy of modern computational clustering methods: is there a clustering method capable of grouping patients from these three datasets into two clinically meaningful clusters? We tried eight different methods, and DBSCAN was the only technique that succeeded in this task.

**Fig. 1:**
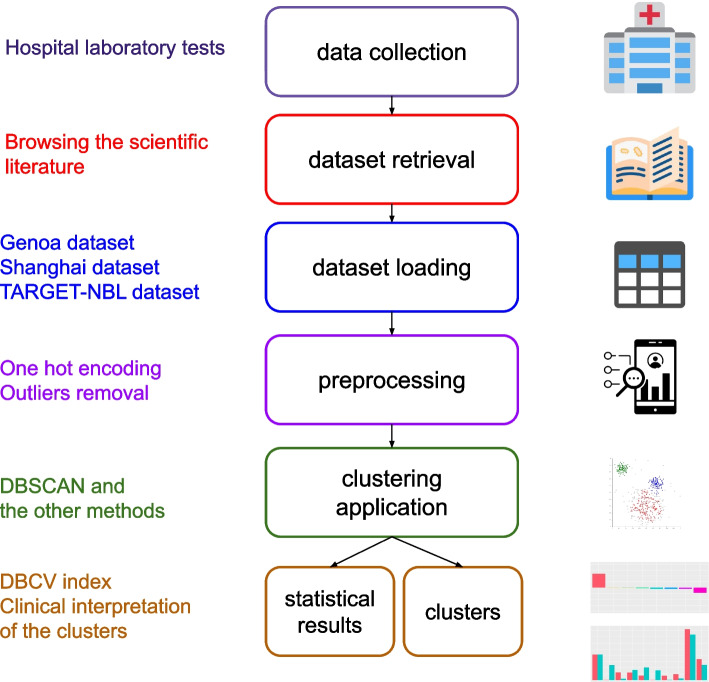
Schematic representation of our study process. The data collection phase was conducted by the original datasets curators in their hospitals [10, 11, 13]. We conducted the dataset retrieval via scientific literature search engines. The steps from dataset loading to statistical results and clusters refer to the computational analysis presented in this study. All the illustration images were released online publicly under a Creative Commons license: the hospital icon from IconScout.com, the book icon from IconScout.com, the table icon from Wikimedia Commons, the barchart icon from IconScout.com, the clusters image from Wikimedia Commons

We organize the rest of the article as follows. After this Introduction, we describe the TARGER-NBL dataset and we outline the Genoa and Shanghai datasets in “Datasets” section. We then briefly describe the clustering computational algorithms employed in “Methods” section and the study results in “Results” section. Finally, we outline a discussion and the main conclusions of this work, including limitations and future developments, in “Discussion” and “Conclusions” sections. We represent all the steps of our study, both the manual ones and the computational ones, in Fig. 1.

## Datasets

In this clustering study, we analyze three independent, open, deidentified datasets of electronic health records: the Genoa dataset [10], the Shanghai dataset [11], and the TARGET-NBL stage 4 dataset [12–14].

We initially conducted a dataset search, by looking for scientific articles on neuroblastoma electronic medical records which included a public dataset in their supplementary information (Fig. 1). We found five public datasets which we cleaned and released in our repository [16, 17] online for free. Among these five datasets, three were too small for any computational analysis, and thus we discarded them: dataCK2018 with 20 patients [22], dataEV2013 with 19 patients [23], and dataYBC2019 with 7 patients [24].

The only two datasets with a sufficient number of patients were *dataBB2013* containing data from 121 patients, which we have renamed here the Genoa dataset, and *dataYM2018* consisting of data from 169 patients, which we have renamed here the Shanghai dataset.

The Genoa dataset was collected at Gaslini hospital in Genoa (Italy, EU) between 1990 and 2004 [10], and contains data from 121 single patients, each having 11 clinical variables The Shanghai dataset was collected at Children’s Hospital of Fudan University in Yangpu (Shanghai, China) between 2010 and 2015 [11], and consists of data of 169 single patients. Each patient profile includes 13 clinical features. We described the Genoa dataset and the Shanghai dataset precisely in the [16] article.

The TARGET-NBL stage 4 dataset contains data from 91 patients collected at the Children’s Hospital of Philadelphia (Pennsylvania, USA) and at other hospitals in the USA, gathered over several years, primarily from 2005 to 2017. This dataset consists of 16 clinical variables, some having missing values. We report a quantitative description of this dataset in Table 1 and Table 2.

**Table 1:** TARGET-NBL stage-4 patients dataset, quantitative characteristics of the binary features. NA: missing values. More information on this dataset can be found in [12–14]

Feature	Meaning	Value	#	%
Differentiating grade	undiff. or poorly diff.	0	72	79.121
Differentiating grade	differentiated	1	6	6.593
Differentiating grade	missing	NA	13	14.286
Histology	unfavorable	0	85	93.407
Histology	favorable	1	2	2.198
Histology	missing	NA	4	4.395
*MYCN* amplification	false	0	68	74.725
*MYCN* amplification	true	1	23	25.275
Sex	woman	0	36	39.560
Sex	man	1	55	60.440
Vital status	alive	0	38	41.758
Vital status	dead	1	53	58.242

**Table 2:** TARGET-NBL stage-4 patients dataset, quantitative characteristics of the numerical features. First event: 0 event, 1 progression, 2 relapse, 3 death. std. dev.: standard deviation. MKI: Mitotic Karyorrhectic Index. Percent Necrosis: percentage of dead tumor cells within the cancerous tissue, which can be an important factor in the pathology and prognosis of the disease. Percent Tumor: percentage of tumor cells present in a biopsy or surgical specimen compared to normal cells. Percent Tumor vs Stroma: percentage of tumor cells to stromal cells within a tumor sample. The stroma is the supportive tissue surrounding the tumor cells, which includes connective tissue, blood vessels, and immune cells. Ploidy Value: number of sets of chromosomes in the tumor cells. Neuroblastoma can be classified as either diploid (normal chromosome number) or aneuploid (abnormal chromosome number). Aneuploid tumors often indicate a more aggressive disease and are associated with poorer outcomes. In contrast, diploid tumors may have a better prognosis. Supenhancer group: 0 *ATRX*, 1 *MES*, and 2 *MYCN*, as defined in [12]. More information on this dataset can be found in [12–14]

Feature	Median	Mean	Range	Std. dev.	#NAs
Age at diagnosis days	1328.000	1618.813	[550, 6021]	1112.958	
Event Free Survival Time in Days	709.000	1130.341	[87, 4948]	1030.418	
First event	2.000	1.746	[0, 3]	0.842	28
MKI	1.000	1.000	[0, 2]	0.799	18
Overall Survival Time in Days	1330.000	1498.923	[87, 4948]	1050.606	
Percent Necrosis	10.000	16.383	[0, 90]	18.583	27
Percent Tumor	80.000	71.098	[5, 97.5]	23.518	25
Percent Tumor vs Stroma	80.000	70.841	[5, 98]	23.076	28
Ploidy Value	1.030	1.218	[1, 3]	0.377	
Superenhancer group	0.000	0.681	[0, 2]	0.880	
Years from diagnosis to last follow-up	4.000	4.055	[0, 14]	2.911	

Our clustering study has been possible because the original curators of these datasets decided to release these datasets publicly online without restrictions, after obtaining the consent from the Institutional Review Board (IRB) of their hospital, following the open science best practices [25, 26].

## Methods

### Preprocessing

We initially applied the clustering methods to the three datasets without removing any outliers from them. This way, we obtained satisfactory results on the Genoa and on the TARGET-NBL datasets, but not on the Shanghai dataset. On the Shanghai original dataset, we had to remove the most different 41% outliers: we computed the average record value for all the patients, we ranked them in descending order, and then we removed the top 20% and the bottom 20%. By doing so, we removed 69 outliers and ultimately obtained valuable clustering results. We used one-hot encoding to handle the *first event* variable of this dataset; all the other features are numeric and therefore do not need this step (Fig. 1).

Given the high heterogeneity of this dataset [11], which is further amplified by the general heterogeneity of neuroblastoma data [27–29], it was necessary to remove 41% of data points from the Shanghai dataset. This step allowed DBSCAN to identify relevant clusters among the remaining data. Without this adjustment, DBSCAN assigns all data points to the noise clusters.

### Clustering algorithms

Before applying the clustering algorithms, we decided to set the number of clusters to two, similar to what has been done in other neuroblastoma studies involving clustering analyses [30–34]. In our work, we employed several clustering algorithms using the scikit-learn Python library [35], each based on distinct principles and requiring specific hyperparameter tuning for optimal performance.

*k*-Means [36] is a popular clustering method that partitions data into a predefined number of clusters by minimizing within-cluster variance. The primary parameter for this algorithm is the number of clusters, which must be specified in advance. *k*-Means assigns points to clusters iteratively until convergence. Spectral Clustering [37] leverages the eigenvalues of a similarity matrix to reduce dimensionality before applying clustering. Key parameters include the number of clusters, the kernel coefficient $\gamma$ gamma for the radial basis function (RBF) kernel, and the number of eigenvectors used in the clustering process. This method is particularly effective for non-linear data separations.

Agglomerative Clustering [38], including Ward’s method [39] and other linkage strategies such as complete, average, and single linkage, performs hierarchical clustering by successively merging pairs of clusters. Important parameters include the number of clusters, the type of linkage criterion used, and the distance metric applied, such as Euclidean, Manhattan, or cosine. BIRCH (Balanced Iterative Reducing and Clustering using Hierarchies) [40] builds a clustering feature tree, making it suitable for large datasets. Its key parameters include the number of clusters, the threshold for forming sub-clusters, and the branching factor, which determines the maximum number of sub-clusters within a node.

Gaussian Mixture [41] models data as a combination of multiple Gaussian distributions, allowing for flexible cluster shapes. Essential parameters include the number of components (clusters) and the covariance type, which can be full, tied, diagonal, or spherical, reflecting different assumptions about the cluster shapes.

DBSCAN (Density-Based Spatial Clustering of Applications with Noise) [42] forms clusters based on dense regions of points. Its two key parameters are the maximum distance between points in a neighborhood and the minimum number of points required to form a dense cluster. It can identify clusters of varying shapes and sizes while labeling outliers.

Affinity Propagation [43] identifies clusters by exchanging messages between points until convergence. The damping parameter, which controls the extent of message propagation, is crucial for avoiding oscillations during the clustering process.

MeanShift [44] locates high-density regions by shifting data points toward density peaks. The bandwidth parameter, which determines the size of the search window, is critical for accurate clustering.

In most of these algorithms (for example, *k*-Means), the numberof clusters is specified a priori (in our case, 2). For algorithms where this parameter cannot be set in advance (for example, DBSCAN), we disregarded parameter combinations that did not yield the desired number of clusters. We report the values of all the optimized hyperparameters for these algorithms in Table 3.

We selected eight of the most common clustering methods employed in the health informatics literature, for which an open source Python implementation is available [35]. Among these algorithms, DBSCAN is known to be particularly effective in biomedical datasets [45, 46]. This step refers to the clustering application phase in the flowchart of Fig. 1.

### Evaluation metric

Finally, these clustering methods have been evaluated using the Density-Based Clustering Validation (DBCV) metric [47] (with the Felipe Alves Siqueira’s Python implementation [48]), which assesses clustering quality by balancing density-based validation criteria. The code iterates through various parameter combinations, aiming to maximize clustering performance.

Here we decided not to utilize common metrics for clustering internal assessments (such as Silhouette coefficient [49], Davies-Bouldin index [50], Calinski-Harabasz index [51], Dunn index [52], Shannon entropy [53], and Gap statistic [54]) because these indexes work only on convex-shaped clusters, and not on concave-shaped clusters. DBSCAN, in fact, can produce both convex or concave clusters, which can be correctly assessed by the DBCV score.

We decided to employ several density-based clustering algorithms and to use the DBCV index for evaluation because of their capability to handle non-convex or irregularly-shaped clusters.

We then studied the clusters assigned by the top performing method, DBSCAN, which is also the only method which produced sufficient results (Fig. 1).

**Table 3:** Optimized hyperparameters for the tested algorithms on the two datasets analyzed. These hyperparameters refer to the functions implemented in scikit-learn Python library [35]

**Genoa dataset**	
Affinity Propagation: damping = 0.97465	
Agglomerative Clustering: linkage = single, metric = euclidean	
BIRCH: branching factor = 4, threshold = 0.88862	
DBSCAN: epsilon = 0.792016, min samples = 8	
Gaussian Mixture: covariance type = full	
* k*-Means: init = k-means++, n init = auto, max iter = 300, algorithm = lloyd	
Spectral Clustering: gamma = 0.88862, n components = 2	
Ward: linkage = ward, metric = euclidean	
**Shanghai dataset**	
Agglomerative Clustering: linkage = single, metric = L1	
BIRCH: branching factor = 2, threshold = 0.888623	
DBSCAN: epsilon = 0.526646, min samples = 4	
Gaussian Mixture: covariance type = diag	
* k*-Means: init = k-means++, n init = auto, max iter = 300, algorithm = lloyd	
Spectral Clustering: gamma = 0.388815, n components = 2	
Ward: linkage = ward, metric = euclidean	
**TARGET-NBL stage-4 dataset**	
Agglomerative Clustering: linkage = average, metric = cosine	
BIRCH: branching factor = 4, threshold = 0.888624	
DBSCAN: epsilon = 0.35019, min samples = 4	
Gaussian Mixture: covariance type = full	
* k*-Means: init = k-means++, n init = auto, max iter = 300, algorithm = lloyd	
Spectral Clustering: gamma = 0.0001, n components = 9	
Ward: linkage = ward, metric = euclidean	

## Results

We applied all the unsupervised clustering methods described in the previous section to the three datasets described in the Datasets section. We optimized the hyperparameters of the algorithms (Table 3) and tested the algorithms’ configurations through the DBCV index [47].

**Fig. 2:**
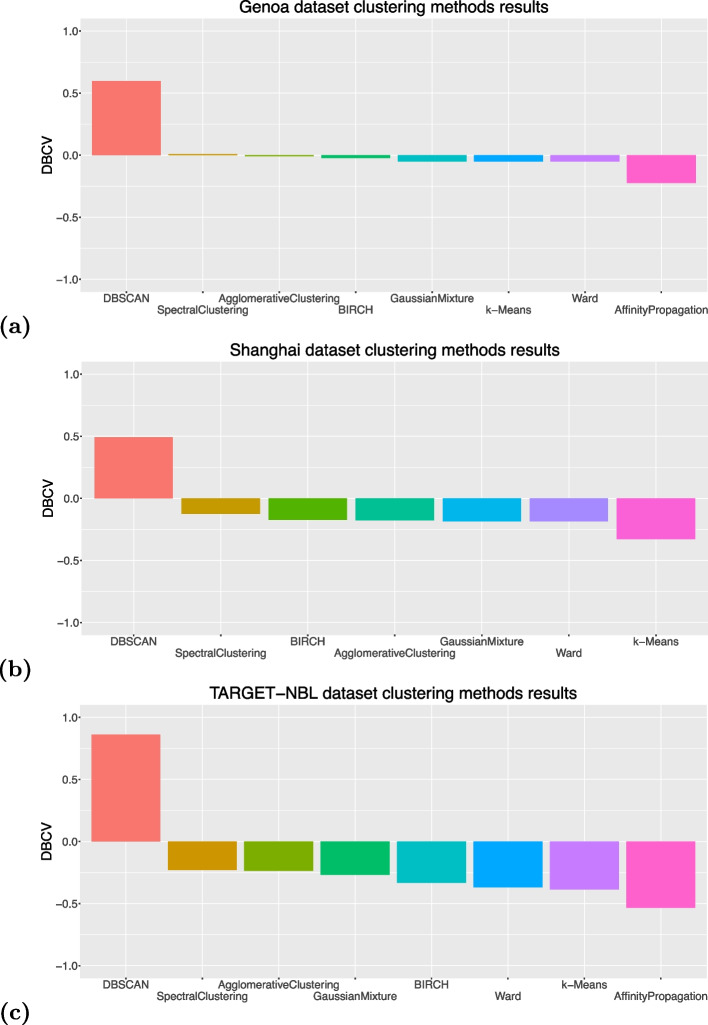
Clustering results obtained by the clustering algorithms on the two dataset. **a** Upper image: Genoa dataset. **b** Mid image: Shanghai dataset. **c** Bottom image: TARGET-NBL dataset. We employed the DBCV (Density-Based Clustering Validation) index for clustering internal assessment. DBCV index interval: $[-1;+1]$, with $-1$ meaning worst possible clustering and $+1$ meaning perfect clustering. We also tried Mean-Shift algorithm on the Genoa and TARGET-NBL datasets, Affinity Propagation on the Genoa and Shanghai datasets, but our scripts generated $-\infty$ for these cases, probably for some code implementation bugs

All the algorithms obtained negative values of DBCV, except DBSCAN which attained $DBCV = +0.5968$ on the Genoa dataset, $DBCV = +0.49256$ on the Shanghai dataset, and $DBCV = +0.86032$ on the TARGET-NBL (Fig. 2). The worst and minimum value of DBCV is $-1$, while the best and maximum value of DBCV is $+1$. Some of the algorithms (Mean-Shift algorithm on the Genoa dataset and Affinity Propagation on both datasets) achieved $DBCV = -\infty$, indicating the possible presence of a technical bug in the implementation of the Python DBCV package [48].

The top performing DBSCAN method identified two clusters in each of the three datasets having different sizes (Table 4).

**Table 4:** Composition of the clusters identified by DBSCAN. removed: number of patients removed during preprocessing. Each cell represents a number of patients

			DBSCAN		
Dataset	Size	Removed	Noise cluster	Cluster 0	Cluster 1
Genoa dataset	121		33	76	12
Shanghai dataset	169	69	88	6	6
TARGET-NBL stage-4 dataset	91		82	5	4

**Fig. 3:**
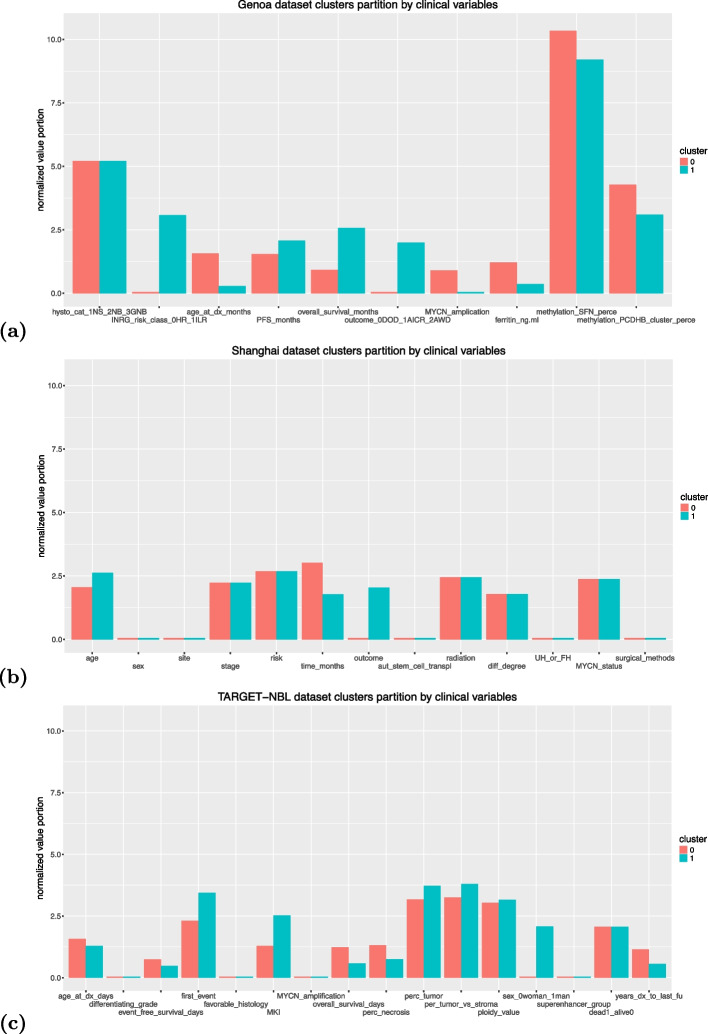
Partition of the clinical features among the two clusters identified by DBSCAN. **a** Top image: representation of the normalized values of the clinical variables of the Genoa dataset in the subset of patients of the 0 cluster (red bars) and in the subset of patients of the 1 cluster (green bars). **b** Mid image: representation of the normalized values of the clinical variables of the Shanghai dataset in the subset of patients of the 0 cluster (red bars) and in the subset of patients of the 1 cluster (green bars). **c** Bottom image: representation of the normalized values of the clinical variables of the TARGET-NBL dataset in the subset of patients of the 0 cluster (red bars) and in the subset of patients of the 1 cluster (green bars). Each bar represents the average value for that specific factor for that specific cluster. We listed the meaning of the clinical variables in “Datasets” section and in [16]

We then scrutinized the results obtained by DBSCAN and observed the feature partitions of the patient clusters identified by this algorithm (Fig. 3a).

In the Genoa dataset results, three clinical features completely partition the dataset patients into two clusters: INRG risk classification, outcome, and *MYCN* amplification. The INRG risk classification feature clearly partitions the data into cluster 0 for high risk and into cluster 1 for intermediate or low risk. Similarly, the outcome variable assigns all the *dead of disease* patients into cluster 0, and all the patients with the *alive in complete remission* or *alive with disease* in cluster 1. Also the *MYCN* gene amplification clearly separates the patients into the two clusters: all patients having *MYCN* amplification were assigned to the cluster 0, and all the patients without were set to cluster 1.

Some other clinical variables showed average differences among the two clusters (age at diagnosis, progression free survival months, overall survival months, ferritin), but did not partition the patients into two completely separated groups (Fig. 3a). These results show the partitioning power of the three clinical variables INRG risk classification, outcome, and *MYCN* gene amplification.

Regarding the Shanghai dataset, a few variables indicated differences in the composition of the two clusters of patients: age, months time of overall survival and outcome (Fig. 3b). Among these three medical variables, only outcome completely separated the patients into two clusters. All the alive patients and all the patients that were lost during follow-up were assigned to cluster 0, and all the patients dead of disease were assigned to cluster 1.

Only the sex variable completely discriminated the two clusters of patients in the TARGET-NBL stage-4 dataset (Fig. 3c): all the women were included in cluster 0, while all the men were put in cluster 1. Other variables, however, indicated a clear separation between the two clusters. DBSCAN assigned to cluster 0 the majority of patients with more years from diagnosis to last follow-up, more overall survival days, and higher percentage of necrosis. The patients assigned by DBSCAN to cluster 1, instead, had more severe first neuroblastoma events and a higher MKI score. The patients of the two clusters had several other minor differences in other clinical features (age at diagnosis, percentage of tumor, percentage of tumor versus stroma, and ploidy value), but we consider these differences irrelevant (Fig. 3c).

For all the three datasets analyzed, DBSCAN assigned some patients to the noise $-1$ cluster: 33 patients out of 121 in the Genoa dataset, 88 out of 100 in the Shanghai dataset (where 69 patients were already removed during the preprocessing phase), and 82 out of 91 in the TARGET-NBL dataset. The algorithm considered these data points as outliers and inserted them into no cluster.

**Table 5:** Results obtained by DBSCAN on ten subsampled datasets. DBSCAN refers to the optimized hyperparameters listed in Table 3. DBCV index interval: $[-1;+1]$, the higher the better

	Average	Standard
Dataset	DBCV index	Deviation
Genoa dataset	0.858	±0.0024
Shanghai dataset	0.779	±0.0212
TARGET-NBL stage-4 dataset	0.941	±0.0006

### Tests of robustness

To verify the robustness of the results we obtained, we performed a subsampling analysis without repetitions. For each of the ten iterations, we randomly selected 90% of the data points, applied DBSCAN, and saved the results measured though the DBCV index. We reported these results in Table 5. These outcomes confirm the robustness of our approach, indicating even an average improvement compared to the results obtained on the whole datasets.

## Discussion

Neuroblastoma is a rare type of childhood cancer characterized by significant heterogeneity in its clinical presentation and in the underlying biological mechanisms driving its onset and development [9]. Clinical parameters such as age, stage, and MYCN amplification status are used at diagnosis to assign a risk group. Risk assignment is used to determine the most appropriate treatment according to international guidelines, such as the INRG pretreatment risk assignment system [55]. Despite multimodal treatments, disease outcomes remain poor for high-risk patients.

The scientific community has conducted numerous studies in recent years, proposing new treatments, prognostic factors, and therapeutic targets [5, 9, 56–59]. Supervised and unsupervised approaches, such as classification and clustering, are two most commonly encountered knowledge-discovery techniques [60]. Supervised approaches have been widely reported in the literature to analyze multi-omics data, EHRs and medical images to accurately stratify patients with neuroblastoma and to predict prognosis [61]. However, to the best of our knowledge, unsupervised approaches have not previously been reported for neuroblastoma EHRs analyses. The problem of clustering in general deals with partitioning a data set consisting of n points embedded in *m*-dimensional space into *k* distinct set of clusters of similar data points [60]. Traditional clustering algorithms use distance metrics such as Euclidean distance to assess similarity among data points. Although these metrics are suitable for features with purely numeric values, they fail to capture the similarity of data elements when attributes are categorical or mixed [60]. Clustering mixed data sets into meaningful groups is a well-known challenging task [60]. Discretization and dummy coding are straightforward and intuitive methods for creating a homogeneous dataset consisting solely of categorical data, enabling the application of classical techniques. However, these methods can distort the original data, potentially introducing bias. In the literature, a variety of clustering algorithms have been specifically designed to handle mixed data [62].

Previously published studies exploring appropriateness of unsupervised machine-learning methods for “heterogeneous” or “mixed” data used simulated and large real world datasets [62]. Pediatric datasets represented emblematic use cases for testing unsupervised clustering methods on mixed data because pediatric disease can be heterogeneous and the number of patients enrolled in the study rarely exceeds one thousand patients. The three datasets used in the present retrospective study are publicly available [10–14].

Datasets are composed of mixed EHRs features covering patients with neuroblastoma of all risk groups, as is the case of the Genoa and Shanghai datasets, as well as, the subset of high-risk patients, as is the case of the TARGET-NBL dataset. The dataset with the highest number of patients is the Shanghai dataset with 169 patients. Therefore, feasibility of unsupervised machine-learning methods for small, heterogeneous and mixed datasets remains to be demonstrated. Our analyses demonstrated that DBSCAN was the unique method able to identify clusters with a clear segregation in the NB datasets. Heterogeneity of the datasets brought the DBSCAN algorithm to cluster a large number of patients of the Shanghai and TARGET-NBL datasets into the noise group. Previous studies have highlighted the effectiveness of a modified version of the DBSCAN algorithm for mixed data analysis, but none have tested the method on real world pediatric datasets [63].

Our density-based spatial clustering analysis assigned significant portions of the Genoa dataset to the two clusters (63% to the first cluster and 10% to the second cluster), but only few patients in the other two datasets. In fact, for the TARGET-NBL dataset only 5% of patients were placed in the first cluster and only 4% of them were assigned into the second cluster, leaving 91% of patients in the noise cluster. For the Shanghai dataset, we initially used all the dataset, but no method could find any cluster in it. So we had to remove the top 20% and the bottom 20% outliers and to work only on 100 patients out of 69. BSCAN placed 6 of these patients’ profiles in the first cluster and 6 in the second cluster, assigning the remaining 88 to the noise cluster. For this dataset, we assigned 92% of patients to no cluster.

We recognize that these clusters are small compared to those in other health informatics studies involving cluster analysis. However, with a rare disease such as neuroblastoma, where datasets are rare, data are small and so are the number of patients, we consider this result to be relevant for the medical significance of the clusters identified. In a landscape where seven traditional clustering algorithms found nothing, at least DBSCAN was able to find thus: even if small, these clusters make clinical sense, and therefore confirm the clustering capability of DBSCAN.

Moreover, neuroblastoma data are known to be extremely heterogeneous [27–29], making them difficult to analyze and process, especially in an unsupervised scenario. The consequence of this heterogeneity is that the the noise clusters identified by DBSCAN have a huge size, compared to te data clusters.

We used the pre-treatment risk groups, when available, and the outcome to evaluate the prognostic value of each cluster. Analysis of the main characteristics differentiating clusters revealed a clear association between clusters and prognosis in the Genoa and Shanghai datasets, thereby confirming the feasibility and potential utility of DBSCAN on small, heterogeneous and mixed data analysis.

## Conclusions

Neuroblastoma is a rare cancer that affects around five thousand infants worldwide, and datasets for scientific research on this disease are scarce. In this study, we leveraged three open, unrestricted, public datasets of electronic health records of patients diagnosed with this pediatric cancer to identify clustering methods which can discriminate significant subgroups of patients. To do so, we took advantage of eight different unsupervised clustering methods and of the DBCV metric implemented in Python, and then analyzed the the medical meaning of the clusters identified.

Our results show that DBSCAN paired with DBCV implemented through an open source programming language, applied to open data, can produce significant results and outcomes that have clinical meaning. Health informatics researchers and analysts can now leverage our discoveries and, when conducting a clustering analysis on small dataset of EHRs of neuroblastoma, can choose to use DBSCAN rather than utilizing more traditional techniques *k*-Means and DBCV rather than utilizing more traditional metrics such as Silhouette coefficient. We highlight the fact that DBSCAN is not only the best performing method among the ones that we employed, but it is also the only one that was able to obtain sufficient results, according to the DBCV index.

Moreover, our study stands for its adherence to the principles of open science: we utilized only open source software code (in Python) to analyze only open data (of electronic medical records), and are publishing the results in an open access journal. Anyone with a technological device can read our findings, reuse our software code, and reutilize the datasets we analyzed here.

Regarding limitations, we need to report that unfortunately we employed datasets with many different features: it would have been better to use datasets having all the same variables, but it was impossible. To the best of our knowledge, no other open EHRs datasets than the three we employed exist in the scientific literature nowadays. Moreover, our clustering analysis did not identify any relevant clinical feature that might impact neuroblastoma treatment or research. In the future, we plan to repeat a similar unsupervised computational analysis on medical records’ data of patients with glioblastoma [64] and other diseases.

## Data Availability

The Genoa and Shanghai datasets used in this project are openly, publicly available for free under the CC BY-NC 4.0 license at: https://davidechicco.github.io/neuroblastoma_EHRs_data The TARGET-NBL stage-4 dataset employed in this study is openly, publicly available for free under the CC BY-NC 4.0 license at: https://figshare.com/articles/dataset/Clinical_and_mutational_information_of_analyzed_94_Stage_4_neuroblastoma_cases_in_TARGET_cohort_/13609375 Our Python software code is publicly available at: https://colab.research.google.com/drive/1NJ3HaljQos0JNRjt4DGF-FSEOcSYVO7c?usp=sharing.

## Abbreviations

*ATRX*: Protein-coding gene, ATRX Chromatin Remodeler
BHLH: Basic helix-loop-helix
BIRCH: Balanced iterative reducing and clustering using hierarchies
CSV: Comma-separated values
DBCV: Density-Based Clustering Validation
DBSCAN: Density-based spatial clustering of applications with noise
Dx: Diagnosis
EHRs: Electronic health records
INRG: International Neuroblastoma Risk Group
INSS: International Neuroblastoma Staging System
LDH: Lactic acid dehydrogenase level
MES: Mesenchymal
*MYCN*: Protein-coding *MYCN* Proto-Oncogene, BHLH Transcription Factor
NB, NBL: Neuroblastoma
PCDHB: Protocadherin Beta Cluster
RBF: Radial basis function
RINB: Italian Registry of Peripheral Neuroblastoma
TARGET: Therapeutically Applicable Research to Generate Effective Treatments
